# Supplementary material for: CRUSTY: a versatile web platform for the rapid analysis and visualization of high-dimensional flow cytometry data
Source: Nat Commun. 2023 Sep 4;14:5102. doi: 10.1038/s41467-023-40790-0 (PMC10477295; doi:10.1038/s41467-023-40790-0)
Supplement: Supplementary file 1 — Supplementary Information [file 41467_2023_40790_MOESM1_ESM.pdf]

CRUSTY: a versatile web platform for the rapid analysis and visualization of high-dimensional flow cytometry data

by Simone Puccio[1,2,*,#], Giorgio Grillo[3,*], Giorgia Alvisi[1], Caterina Scirgolea[1], Giovanni Galletti[1,†], Emilia Maria Cristina Mazza [1], Arianna Consiglio[3], Gabriele De Simone[4], Flavio Licciulli[3] and Enrico Lugli[1,#]

[1] Laboratory of Translational Immunology, IRCCS Humanitas Research Hospital, via Manzoni 56, 20089, Rozzano, Milan, Italy

[2] Institute of Genetic and Biomedical Research, UoS Milan, National Research Council, via Manzoni 56, 20089, Rozzano, Milan, Italy

[3] Institute for Biomedical Technologies, National Research Council, via Amendola 122/D, 70126, Bari, Italy

[4] Flow Cytometry Core, IRCCS Humanitas Research Hospital, via Manzoni 56, 20089, Rozzano, Milan, Italy

[†] Present address: School of Biological Sciences, Department of Molecular Biology, University of California San Diego, San Diego, CA, USA.

[#] Corresponding authors: Simone Puccio and Enrico Lugli, Laboratory of Translational Immunology, Via Manzoni 56, Rozzano (MI), Italy, Phone: +39 02 8224 5143, Fax: +39 02 8224 5191. E-mail: enrico.lugli@humanitasresearch.it and simone.puccio@humanitasresearch.it

This document includes Supplementary Table 1

Supplementary Table 1. Comparison of specific features between CRUSTY and similar software packages

**Functions**	**Cytofkit**	**CytoChain**	**CRUSTY**
Automated Data Cleaning		✓	✓
Clustering	✓	✓	✓
Dimensionality reduction	✓	✓	✓
Batch Correction			✓
Sample datasets	✓		✓
Web application		✓	✓
Interactive exploration of results	✓		✓
Code availability	✓		✓
