# Supplementary material for: CRUSTY: a versatile web platform for the rapid analysis and visualization of high-dimensional flow cytometry data
Source: Nat Commun. 2023 Sep 4;14:5102. doi: 10.1038/s41467-023-40790-0 (PMC10477295; doi:10.1038/s41467-023-40790-0)
Supplement: Supplementary file 3 — Reporting Summary [file 41467_2023_40790_MOESM3_ESM.pdf]

nature portfolio

Corresponding author(s): Enrico Lugli

Last updated by author(s): Jul 18, 2023

Reporting Summary

Nature Portfolio wishes to improve the reproducibility of the work that we publish. This form provides structure for consistency and transparency in reporting. For further information on Nature Portfolio policies, see our Editorial Policies and the Editorial Policy Checklist.

Statistics

For all statistical analyses, confirm that the following items are present in the figure legend, table legend, main text, or Methods section.

n/a	Confirmed	
[x]	[ ]	The exact sample size (*n*) for each experimental group/condition, given as a discrete number and unit of measurement
[x]	[ ]	A statement on whether measurements were taken from distinct samples or whether the same sample was measured repeatedly
[x]	[ ]	The statistical test(s) used AND whether they are one- or two-sided<br>*Only common tests should be described solely by name; describe more complex techniques in the Methods section.*
[x]	[ ]	A description of all covariates tested
[x]	[ ]	A description of any assumptions or corrections, such as tests of normality and adjustment for multiple comparisons
[x]	[ ]	A full description of the statistical parameters including central tendency (e.g. means) or other basic estimates (e.g. regression coefficient) AND variation (e.g. standard deviation) or associated estimates of uncertainty (e.g. confidence intervals)
[x]	[ ]	For null hypothesis testing, the test statistic (e.g. *F*, *t*, *r*) with confidence intervals, effect sizes, degrees of freedom and *P* value noted<br>*Give P values as exact values whenever suitable.*
[x]	[ ]	For Bayesian analysis, information on the choice of priors and Markov chain Monte Carlo settings
[x]	[ ]	For hierarchical and complex designs, identification of the appropriate level for tests and full reporting of outcomes
[x]	[ ]	Estimates of effect sizes (e.g. Cohen's *d*, Pearson's *r*), indicating how they were calculated

Our web collection on statistics for biologists contains articles on many of the points above.

Software and code

Policy information about availability of computer code

Data collection: No new data was generated for this study. FCM files are free available and were downloaded from https://flowrepository.org/ with the following id FR-FCM-Z5LE

Data analysis: Pipeline source code and dependences are freely available for download at GitHub: https://github.com/luglilab/Cytophenograph.

Version of package used:
phenograph=1.5.7
flowai=1.28.0
flowcore=2.10.0
matplotlib-base=3.6.3
numba=0.56.4
numpy=1.23.5
python=3.9.16
r-base=4.2.2
pandas==1.3.5
pyvia==0.1.73
scanpy==1.9.1
scipy==1.10.0
scprep==1.2.1

umap-learn==0.5.3

For manuscripts utilizing custom algorithms or software that are central to the research but not yet described in published literature, software must be made available to editors and reviewers. We strongly encourage code deposition in a community repository (e.g. GitHub). See the Nature Portfolio guidelines for submitting code & software for further information.

Data

Policy information about availability of data

All manuscripts must include a data availability statement. This statement should provide the following information, where applicable:

- Accession codes, unique identifiers, or web links for publicly available datasets
- A description of any restrictions on data availability
- For clinical datasets or third party data, please ensure that the statement adheres to our policy

FCM data of CD8+ T cells previously reported in Fig. 1g-1 from Galletti et al. were isolated as in Supplementary Fig1 compensated and bi-exponentially transformed with FlowJo v10.5.0 according to standard procedures and reanalyzed in Crusty. Pre-processed CD8+ FCM data are available at https://flowrepository.org/ with the following id FR-FCM-Z5LE. Source data can be downloaded from the CRUSTY website.

Human research participants

Policy information about studies involving human research participants and Sex and Gender in Research.

Reporting on sex and gender	N/A
Population characteristics	N/A
Recruitment	N/A
Ethics oversight	N/A

Note that full information on the approval of the study protocol must also be provided in the manuscript.

Field-specific reporting

Please select the one below that is the best fit for your research. If you are not sure, read the appropriate sections before making your selection.

☒ Life sciences ☐ Behavioural & social sciences ☐ Ecological, evolutionary & environmental sciences

For a reference copy of the document with all sections, see nature.com/documents/nr-reporting-summary-flat.pdf

Life sciences study design

All studies must disclose on these points even when the disclosure is negative.

Sample size	Sample size was chosen taking into account the means of the target values between the different experimental groups, the standard error and the statistical analyses used. Additionally, the section of sample size was based on previous studies conducted by this laboratory, which allowed statistically valid comparisons.
Data exclusions	No data were excluded. Filtering and quality control of cells is described in the "Pre-processing and quality control" section.
Replication	All the computational analysis were replicated several times (on Linux servers). All attempts at replication were successful.
Randomization	We have reanalyzed an existing dataset without any direct intervention or treatment administration to participants.
Blinding	No new data was generated for this study. All results are based on published data which have been studied in their original publications. Therefore, blinding from investigators is not possible when we reanalyzed the collected data.

Reporting for specific materials, systems and methods

We require information from authors about some types of materials, experimental systems and methods used in many studies. Here, indicate whether each material, system or method listed is relevant to your study. If you are not sure if a list item applies to your research, read the appropriate section before selecting a response.

Materials & experimental systems

n/a	Involved in the study	
☐	☒	Antibodies
☒	☐	Eukaryotic cell lines
☒	☐	Palaeontology and archaeology
☒	☐	Animals and other organisms
☒	☐	Clinical data
☒	☐	Dual use research of concern

Methods

n/a	Involved in the study	
☒	☐	ChIP-seq
☒	☐	Flow cytometry
☒	☐	MRI-based neuroimaging

Antibodies

Antibodies used	Previously described in PMID: 33046887
Validation	Previously described in PMID: 33046887
